# No impact of time to treatment initiation for head and neck cancer in a tertiary university center in 2003, 2008 and 2013

**DOI:** 10.1007/s00405-022-07392-w

**Published:** 2022-04-30

**Authors:** Mussab Kouka, Max Engelhardt, Andrea Wittig, Stefan Schultze-Mosgau, Thomas Ernst, Orlando Guntinas-Lichius

**Affiliations:** 1 Department of Otorhinolaryngology, Jena University Hospital, Am Klinikum 1, 07747 Jena, Germany; 2 Department of Radiotherapy and Radiation Oncology, Jena University Hospital, Jena, Germany; 3 Department of Oromaxillofacial Surgery and Plastic Surgery, Jena University Hospital, Jena, Germany; 4 University Tumor Center, Jena University Hospital, Jena, Germany

**Keywords:** Doctor’s delay, Time to treatment initiation, Head and neck cancer, Treatment delay, Survival

## Abstract

**Background:**

This retrospective study investigated factors influencing time to treatment initiation (TTI) and the influence of TTI on overall survival (OS) of primary head and neck cancer (HNC) patients in cohorts from 2003, 2008 and 2013.

**Methods:**

Two hundred and ninenty seven patients (78.8% men; median age: 62 years) were included. Kaplan–Meier analyses and multivariate Cox regression were performed to investigate OS.

**Results:**

Mean times to treatment initiation (TTI) of 2003, 2008 and 2013 were 17.11 ± 18.00, 30.26 ± 30.08 and 17.30 ± 37.04 days, respectively. TTI for patients with T3/T4 tumors was higher than for T1/T2 (*p = *0.010). In univariable analysis on OS, TTI > 5 days showed lower OS (*p = *0.047). In multivariate analysis, longer TTI had no influence on lower OS [hazard ratio (HR) 1.236; 95% CI 0.852–1.791; *p = *0.264], but male gender [HR 2.342; 95% CI 1.229–4.466; *p = *0.010], increased age [HR 1.026; 95% CI 1.008–1.045; *p = *0.005], M1 [HR 5.823; 95% CI 2.252–15.058; *p = *0.003], hypopharynx tumor [HR 2.508; 95% CI 1.571–4.003; *p <  *0.001] and oral cavity tumor [HR 1.712; CI 1.101–2.661; *p = *0.017]. The year of treatment showed no significant effect on OS.

**Conclusion:**

Median TTI seemed to be very short compared to other studies. There was no clear trend in the impact of TTI on OS from 2003 to 2013.

**Supplementary Information:**

The online version contains supplementary material available at 10.1007/s00405-022-07392-w.

## Introduction

Head and neck cancers (HNCs) are often only diagnosed at an advanced stage [1]. Despite advances in diagnostics and treatment methods, the long-term prognosis remains poor with a 5-year survival rate of 68% [2]. Our main objective was to investigate treatment delays and their impact on overall survival (OS). Time to treatment initiation (TTI) describes delays in treatment of a patient, which mainly occur between the first visit to a doctor and the start of treatment. Delay can be caused, e.g., by waiting times for appointments for specialists, waiting times for diagnostic examination procedures, waiting times for therapies, misdiagnoses and repeated examinations. Therefore, recent studies investigated the impact of TTI together with possibilities of optimization. We prefer the term TTI instead of doctor’s delay. Doctor’s delay may also be used when the disease was not recognized by the doctor immediately. TTI is defined as the number of days between the histopathological diagnosis and the start of primary treatment. The main concern of longer intervals between tumor diagnosis and the start of therapy lies in possible tumor progression and reduced tumor control resulting in more extensive therapy and reduced OS as well as higher health costs [3]. Xiao et al. showed that a longer TTI results in tumor progression and the associated increase in mortality [4]. According to Jensen et al., the median tumor size in HNC doubles within 99 days [5]. Nevertheless, it makes sense to take sufficient time for precise tumor staging, planning and coordinating complex multidisciplinary treatment strategies.

Recent studies from the Unites States have shown that TTI has a significant influence on OS [4, 6]. Delays in TTI can be a significant problem for a patient’s prognosis. In this study, patients with HNC treated in 2003, 2008 and 2013 at a tertiary university hospital were included.

For this purpose, the influence of delays and waiting times on curative treatment in 2003, 2008 and 2013 as well as the impact of TTI on OS were analyzed. In addition, the intervals between examinations and the start of treatment were examined in detail.

## Methods

### Ethical considerations

This study was approved by the Ethics Committee of the Jena University Hospital (IRB No. 3204-07/11). The Ethics Committee waived the requirement for informed consent of the patients because the study had a non-interventional retrospective design and all data were analyzed anonymously.

### Patients

This retrospective study was based on a dataset, which was provided by the Thuringian cancer registry in Jena, Germany. In total, 470 cases were registered in 2003, 2008 and 2013. Patients were excluded if they did not have a HNC, if treatment was performed outside the study period, if no treatment was started, if patients were noted twice and if there was insufficient documentation. Additional clinical data from the patients’ health care records were transferred to the dataset. Patients were divided into three groups according to the year in which they were treated. Histopathological confirmation of cancer was defined for the time of diagnosis. The pathological stages of the primary cancer were recorded using the UICC classification and TNM classification, 7th edition [7]. UICC classification was also used to classify tumor stages.

### Charlson comorbidity index

The Charlson comorbidity index (CCI) is a method of assessing the influence of different comorbidities on a patient's mortality risk [8]. Depending on their relevance, 19 comorbidities are assigned numerical values which are then added together to evaluate the patient’s mortality risk. The dichotomous variable “CCI < median” or “CCI > median” was created to allow statistical analysis.

### Statistical analysis

Descriptive statistics were performed using SPSS Statistics Version 25 (IBM Deutschland GmbH, 71,139 Ehningen, Germany). Absolute and relative frequencies of nominal parameters were calculated using cross tables. For the metric parameters, mean and the standard deviation as well as the median and the range were calculated. Statistical significance was performed using chi-square test for nominal variables. For metric variables, the Kruskal–Wallis test was chosen. Kaplan–Meier calculations were performed to assess the influence of the variables on OS of the patients. The log-rank test was performed to analyze the subgroups for significant differences in survival. *P* ≤ 0.05 was rated as statistically significant. Multivariable analyses were performed using a Cox proportional hazard ratio (HR) with a 95% confidence interval (CI). Variables were taken into account that showed significant differences in survival in the Kaplan–Meier analyses. Variables that fit together in terms of content were jointly investigated, for example, variables of general patient characteristics, tumor or treatment characteristics were included in several Cox models.

## Results

### Patient’s characteristics, tumor characteristics and treatment characteristics

In total, 297 HNC patients were included in the study. Of these, the initial diagnosis was made in 84 patients in 2003, in 108 patients in 2008 and in 105 patients in 2013. As shown in Table 1, men formed the majority of HNC patients (234 men, 78.8%). From 2003 to 2013, the proportion of women increased from 13.1% to 27.6%. The mean age at diagnosis was 61.1 years (5–102 years). The age at diagnosis increased from 2003 to 2013 (*p = *0.024). Mean CCI was 4.8 ± 3.1. The mean duration of therapy from the first day of treatment to the last day of treatment was 86.4 ± 123.9 days (Stage I/II: 81.25 ± 108.61; Stage III/IV: 189.17 ± 141.92). The 2008 cohort showed a larger duration of therapy (*p = *0.042). 41.0% of patients were alcohol drinking, 60.7% were smokers. The 2003 cohort was dominated by smokers (*p <  *0.001) and alcohol drinking patients (*p <  *0.001) compared to the 2008 and 2013 cohorts. More than half of the patients showed advanced T classification (T3/T4: 60%). The largest proportion was in 2013 (T3/T4: 74.3%). In all cohorts, UICC stage III/IV was over presented (2003: 77.8%, 2008: 73.3%, 2013: 85.3%). The distribution of HNC patients was mostly divided between oropharynx (20.5%), larynx (18.5%), oral cavity (12.1%), and hypopharynx (10.4%). Surgery was the most frequently used primary treatment modality (261 patients, 87.9%). In 2013, chemotherapy (*p <  *0.001) and immunotherapy (*p = *0.007) were more frequently used than in the cohorts from 2003 to 2008. Chemo-radiation was more often performed in 2008 (*p = *0.003).

**Table 1:** Patients’ characteristics, histopathology characteristics and treatment characteristics of HNC patients

Parameter	All years		2003		2008		2013		*p*
	*N*	%	*N*	%	*N*	%	*N*	%	
Gender									
Male	234	78.8	73	86.9	85	78.7	76	72.4	0.053
Female	63	21.2	11	13.1	23	21.3	29	27.6	
Alcohol drinking									
Yes	100	41.0	35	79.5	33	34.0	32	31.1	** < 0.001**
No	144	59.0	9	20.5	64	66.0	71	68.9	
Cigarette smoking									
Yes	156	60.7	46	83.6	58	58.6	52	50.5	** < 0.001**
No	101	39.3	9	16.4	41	41.4	51	49.5	
T classification									
T1/T2	29	32.2	10	38.5	10	34.5	9	25.7	0.546
T3/T4	61	67.8	16	61.5	19	65.5	26	74.3	
N classification									
N0	35	34.0	10	33.3	14	41.2	11	28.2	0.504
N1,2,3	68	66.0	20	66.7	20	58.8	28	71.8	
M classification									
M0	95	88.8	30	100.0	30	88.2	35	81.4	0.145
M1	11	10.3	0	0.0	4	11.8	7	16.3	
Cancer stage									
Stage I/II	17	20.7	4	22.2	8	26.7	5	14.7	0.492
Stage III/IV	65	79.3	14	77.8	22	73.3	29	85.3	
Localization									
Cavity of the mouth	36	12.1	10	11.9	18	16.7	8	7.6	0.129
Oropharynx	61	20.5	18	21.4	26	24.1	17	16.2	0.353
Nasopharynx	5	1.7	3	3.6	0	0.0	2	1.9	0.158
Hypopharynx	31	10.4	14	16.7	9	8.3	8	7.6	0.087
Larynx	55	18.5	20	23.8	22	20.4	13	12.4	0.109
Nose	8	2.7	2	2.4	4	3.7	2	1.9	0.704
Parotid gland	11	3.7	2	2.4	5	4.6	4	3.8	0.714
Submandibular gland	2	0.7	0	0.0	1	0.9	1	1.0	0.672
Ear	15	5.1	3	3.6	2	1.9	10	9.5	**0.029**
Facial skin	27	9.1	5	6.0	8	7.4	14	13.3	0.161
Thyroid	6	2.0	1	1.2	2	1.9	3	2.9	0.712
Paranasal sinus	2	0.7	1	1.2	0	0.0	1	1.0	0.552
Esophagus	5	1.7	1	1.2	1	0.9	3	2.9	0.504
Unspecified	33	11.1	4	4.8	10	9.3	19	18.1	**0.011**
Treatment*									
Surgery alone	261	87.9	73	87.0	96	88.9	92	87.6	0.912
Chemotherapy	37	12.5	4	4.8	8	7.4	25	23.8	** < 0.001**
Radiation	113	38.0	37	44.0	38	35.2	38	36.2	0.404
Chemo-radiation	47	15.8	11	13.1	27	25.0	9	85.7	**0.003**
Immunotherapy	8	2.7	0	0	1	0.9	7	0.7	**0.007**

	Mean ± SD	Median, range	Mean ± SD	Median, range	Mean ± SD	Median, range	Mean ± SD	Median, range	*P*
CCI	4.83 ± 3.086	3, 2–16	5.37 ± 3.092	4.5, 2–11	4.62 ± 3.056	3, 2–12	4.62 ± 3.090	3, 2–16	0.088
Age	61.13 ± 13.656	62, 5–102	58.83 ± 11.218	57,38–83	61.0 ± 14.759	63, 21–93	63.06 ± 14.079	63, 5–102	**0.024**
Duration of treatment in days	86.44 ± 123.93	68.5, 1–1330	61.99 ± 77.015	61, 1–378	114.64 ± 170.79	79, 1–1330	76.64 ± 86.250	59, 1–412	**0.042**

*CCI* Charlson comorbidity index, *SD* standard deviation

^*^Sum can be higher than 100%

Significant p-values (p<0.05) in bold

### Time to treatment initiation

**Fig. 1:**
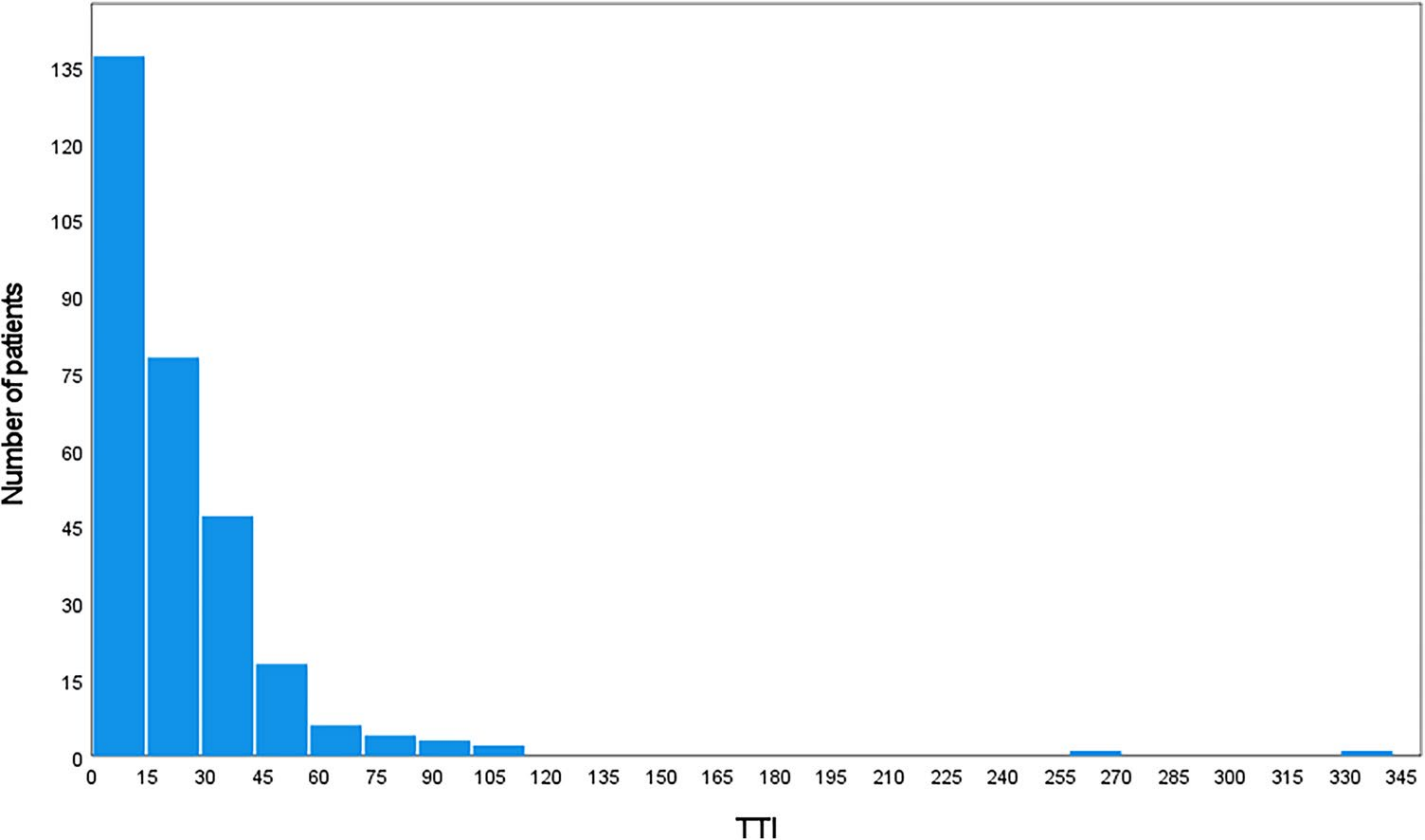
Histogram of time to treatment initiation (TTI) according to absolute number of patients

The median TTI was 16 days (0–339). There was one patient initially declining treatment. This patient came back for treatment about nine month later explaining most of the 339 days of TTI. The frequency distribution is shown in Fig. 1. Table 2 shows the association of TTI with patients’ characteristics, histopathology and treatment. In 2008, men waited significantly longer than women with an average of 33.0 days compared to 20.2 days (*p = *0.023). For men, treatment delay was significantly higher in 2008 than in the other two years (*p <  *0.001). For alcohol drinking patients (26.8 ± 31.7 days), TTI was significantly higher than for non-alcohol drinking patients (21.7 ± 33.5 days, *p = *0.018). Alcohol drinking patients (*p <  *0.001) and non-alcohol drinking patients (*p <  *0.001) showed significantly higher waiting time in 2008 than in 2003 and 2013. TTI (26.5 ± 38.0 days) was for smokers significantly higher than for non-smokers (18.5 ± 19.1 days, *p = *0.029) and showed higher waiting time in 2008 than in 2003 and 2013 (*p <  *0.001, *p <  *0.001). In 2013, a higher CCI showed a longer TTI (24.2 ± 50.4 days) than a lower CCI (11.1 ± 15.9 days, *p = *0.046). Considering the UICC classification, patients with a higher stage (stage III/IV) waited significantly longer than patients with a lower stage (stage I/II, *p = *0.021) only in 2008. HNC patients with oropharynx (*p = *0.047), hypopharynx (*p = *0.020) and esophagus (*p <  *0.001) tumor had significantly longer waiting times than patients with other localization. Patients with HNC of the parotid gland (*p = *0.003), ear (*p = *0.027), facial skin (*p <  *0.001) and unspecified HNC (*p = *0.002) had a shorter TTI than patients with other localizations. TTI was longer in most of the localizations in 2008 than in 2003 and 2013 (mean TTI 22.0 ± 30.7, *p <  *0.001). Considering treatment characteristics, average TTI of surgery was 17.9 ± 26.3 days. The longest TTI for surgery was seen in 2008 with 30.3 ± 30.1 days, which was longer than in 2003 and 2013 (*p <  *0.001). TTI for chemo-radiation was about twice as long as for surgery with an average TTI of 45.77 ± 42.4 days. The TTI for chemo-radiation did not vary significantly between the three cohorts (*p = *0.137). In conclusion, HNC patients of 2008 had longer time lags until treatment initiation compared to HNC patients of 2003 and 2013.

**Table 2:** Time to treatment initiation (TTI) in relation to patients’ characteristics, histopathology characteristics and treatment characteristics of HNC patients

Parameter	All years		2003		2008		2013		*p*
	Mean ± SD	Median, range	Mean ± SD	Median, range	Mean ± SD	Median, range	Mean ± SD	Median, range	
Total waiting time (days) in relation to characteristic									
Gender									
Male	21.79 ± 25.456	17, 0–268	17.16 ± 17.128	15, 0–88	32.98 ± 32.400	26, 0–268	13.72 ± 18.100	10, 0–105	** < 0.001**
Female	22.59 ± 45.271	11, 0–339	16.73 ± 24.017	10, 0–87	20.22 ± 16.091	21, 0–60	26.69 ± 63.978	4, 0–339	0.176
* p*	0.113		0.524		**0.023**		0.920		
Alcohol drinking									
Yes	26.85 ± 31.710	21, 0–268	22.66 ± 19.872	17, 0–88	40.76 ± 44.786	31, 6–268	17.09 ± 19.587	12, 0–76	** < 0.001**
No	21.67 ± 33.538	15, 0–339	11.22 ± 12.498	9, 0–40	27.53 ± 19.853	24.5, 0–91	17.72 ± 43.173	4, 0–339	** < 0.001**
* p*	**0.018**		0.054		**0.038**		0.141		
Cigarette smoking									
Yes	26.46 ± 38.014	19, 0–339	20.09 ± 18.687	15, 0–88	33.47 ± 37.122	26, 0–268	24.29 ± 49.460	12, 0–339	** < 0.001**
No	18.51 ± 19.104	15, 0–78	13.89 ± 12.374	13, 0–36	29.34 ± 18.902	26, 0–78	10.63 ± 16.006	0, 0–61	** < 0.001**
* p*	**0.029**		0.432		0.963		**0.013**		
Age									
< Median	19.68 ± 19.387	15, 0–104	18.66 ± 21.091	12, 0–88	27.45 ± 17.831	26, 0–104	12.07 ± 15.708	5, 0–58	** < 0.001**
> Median	24.26 ± 38.768	16, 0–339	14.45 ± 10.667	15, 0–43	32.77 ± 37.836	26, 0–268	21.23 ± 46.885	7.5, 0–339	** < 0.001**
* p*	0.747		0.978		0.885		0.645		
CCI									
< Median	21.33 ± 28.055	16, 0–268	19.38 ± 21.357	15, 0–88	32.03 ± 35.787	26, 0–268	11.05 ± 15.912	0, 0–76	** < 0.001**
> Median	22.58 ± 33.115	16, 0–339	15.56 ± 15.349	12.5, 0–79	28.12 ± 21.473	24, 0–104	24.18 ± 50.412	10.5, 0–339	**0.001**
* p*	0.739		0.476		0.527		**0.046**		
T classification									
T1/T2	22.34 ± 21.309	16, 0–105	14.00 ± 11.025	11.5, 0–35	25.80 ± 16.144	17.5, 11–58	27.78 ± 32.003	16, 3–105	0.252
T3/T4	36.20 ± 37.581	28, 0–268	33.25 ± 23.029	26, 13–88	53.42 ± 56.944	36, 13–268	25.42 ± 19.621	17, 0–76	**0.012**
* p*	**0.010**		**0.011**		**0.035**		0.678		
N classification									
N0	29.31 ± 44.065	16, 1–268	15.00 ± 11.096	13, 1–40	45.21 ± 65.923	31.5, 11–268	22.09 ± 15.010	16, 3–45	0.077
N1.2.3	28.71 ± 24.702	21.5, 0–105	28.85 ± 23.118	21, 0–88	38.05 ± 23.578	27.5, 16–104	21.93 ± 25.191	13, 0–105	**0.004**
* p*	0.488		0.067		0.431		0.553		
M classification									
M0	28.91 ± 33.023	20, 0–268	23.03 ± 21.129	16, 0–88	39.90 ± 47.273	26, 11–268	24.51 ± 23.896	16, 0–105	**0.014**
M1	26.09 ± 26.082	29, 0–91	–	–	49.25 ± 28.076	3.5, 31–91	12.86 ± 13.335	10, 0–33	**0.018**
* p*	0.752		–		0.148		0.295		
Cancer stage									
Stage I/II	24.41 ± 24.470	16, 1–105	14.00 ± 10.392	15, 1–25	22.63 ± 12.994	15.5, 11–44	35.60 ± 41.801	16, 3–105	0.726
Stage III/IV	33.52 ± 36.754	26, 0–268	25.00 ± 21.422	17, 0–88	51.05 ± 53.430	35.5, 13–268	24.34 ± 19.202	17, 0–76	**0.003**
* p*	0.168		0.365		**0.021**		0.981		
Localization									
Cavity of the mouth									
Yes	24.19 ± 18.919	25, 0–88	20.70 ± 27.390	9.5, 0–88	26.28 ± 9.234	27.5, 11–43	23.88 ± 24.275	20, 0–75	0.186
No	21.65 ± 31.949	15, 0–339	16.62 ± 16.549	14, 0–87	31.06 ± 32.677	26, 0–268	16.76 ± 37.943	7, 0–339	** < 0.001**
* p*	0.071		0.803		0.843		0.120		
Oropharynx									
Yes	22.54 ± 16.646	19, 0–79	18.72 ± 21.997	12.5, 0–79	27.42 ± 12.410	26, 7–58	19.12 ± 14.722	17, 0–54	**0.020**
No	12.81 ± 33.356	15, 0–339	16.67 ± 16.919	15, 0–88	31.16 ± 33.829	26, 0–268	16.95 ± 39.997	3.5, 0–339	** < 0.001**
* p*	**0.047**		0.806		0.768		**0.022**		
Nasopharynx									
Yes	27.20 ± 16.947	31, 9–45	16.67 ± 12.423	10, 9–31	-	-	43.00 ± 2.828	43, 41–45	0.083
No	21.87 ± 30.843	16, 0–339	17.12 ± 18.229	13, 0–88	30.26 ± 30.082	26, 0–268	16.81 ± 37.227	7, 0–339	** < 0.001**
* p*	0.252		0.828		-		**0.038**		
Hypopharynx									
Yes	25.68 ± 16.668	21, 0–62	18.50 ± 9.354	17, 0–36	37.11 ± 15.496	39, 14–62	25.38 ± 21.967	19, 0–61	**0.027**
No	21.53 ± 31.879	15, 0–339	16.83 ± 19.308	10.5, 0–88	29.64 ± 31.044	26, 0–268	16.64 ± 38.020	7, 0–339	** < 0.001**
* p*	**0.020**		0.130		0.062		0.059		
Larynx									
Yes	26.69 ± 37.637	19, 0–268	14.00 ± 10.950	10, 0–35	43.45 ± 54.082	30, 1–268	17.85 ± 14.058	12, 0–46	**0.001**
No	20.88 ± 28.816	15, 0–339	18.08 ± 19.664	14, 0–88	26.88 ± 18.971	25, 0–104	17.23 ± 39.270	4.5, 0–339	** < 0.001**
* p*	0.070		0.744		0.091		0.072		
Nose									
Yes	16.13 ± 14.904	12,5, 0–42	12.50 ± 3.536	12.5, 10–15	26.00 ± 14.213	26.5, 9–42	0.00 ± 0.000	0, 0–0	0.103
No	22.12 ± 30.973	16, 0–339	17.22 ± 18.202	13, 0–88	30.42 ± 30.552	26, 0–268	17.64 ± 37.325	8, 0–339	** < 0.001**
* p*	0.630		0.918		0.974		0.137		
Parotid gland									
Yes	5.55 ± 8.299	0, 0–23	9.00 ± 12.728	9, 0–18	8.60 ± 9.127	7, 0–23	0.00 ± 0.000	0, 0–0	0.112
No	22.59 ± 31.026	16, 0–339	17.30 ± 18.120	13, 0–88	31.31 ± 30.363	26, 0–268	17.99 ± 37.612	9, 0–339	** < 0.001**
* p*	**0.003**		0.436		**0.007**		**0.034**		
Submandibular gland									
Yes	25.50 ± 36.062	25.5, 0–51	–	–	51.00 ± 0.000	51, 51–51	0.00 ± 0.000	0, 0–0	0.317
No	21.94 ± 30.683	16, 0–339	17.11 ± 18.000	13, 0–88	30.07 ± 30.155	26, 0–268	17.47 ± 37.183	7.5, 0–339	** < 0.001**
* p*	0.974		–		0.194		0.296		
Ear									
Yes	15.20 ± 26.902	2, 0–78	11.67 ± 15.044	4, 2–29	78.00 ± 0.000	78, 78–78	3.70 ± 6.929	0, 0–20	**0.025**
No	22.32 ± 30.840	16, 0–339	17.31 ± 18.148	13, 0–88	29.36 ± 29.630	26, 0–268	18.74 ± 38.624	10, 0–339	** < 0.001**
* p*	**0.027**		0.673		**0.023**		**0.037**		
Facial skin									
Yes	10.33 ± 17.863	0, 0–67	17.80 ± 14.132	19, 1–39	20.13 ± 26.787	6, 0–67	2.07 ± 6.032	0, 0–22	**0.003**
No	23.12 ± 31.435	17, 0–339	17.06 ± 18.289	13, 0–88	31.07 ± 30.304	26, 0–268	19.65 ± 39.227	10, 0–339	** < 0.001**
* p*	** < 0.001**		0.576		0.080		**0.001**		
Thyroid									
Yes	7.83 ± 8.565	6.5, 0–21	21.00 ± 0.000	21, 21–21	6.50 ± 6.364	6.5, 2–11	4.33 ± 7.506	0, 0–13	0.294
No	22.25 ± 30.880	16, 0–339	17.06 ± 18.104	13, 0–88	30.71 ± 30.179	26, 0–268	17.69 ± 37.506	7.5, 0–339	** < 0.001**
* p*	0.104		0.445		0.057		0.358		
Paranasal sinus									
Yes	19.00 ± 26.870	19, 0–38	0.00 ± 0.000	0, 0–0	-	-	38.00 ± 0.000	38, 38–38	0.317
No	21.98 ± 30.716	16, 0–339	17.31 ± 18.009	13, 0–88	30.26 ± 30.082	26, 0–268	17.11 ± 37.166	7, 0–339	** < 0.001**
* p*	0.885		0.126		-		0.176		
Esophagus									
Yes	79.60 ± 27.519	87, 44–105	87.00 ± 0.000	87, 87–87	104.00 ± 0.000	104, 104–104	69.00 ± 31.953	58, 44–105	0.766
No	20.97 ± 29.787	15, 0–339	16.27 ± 16.360	13, 0–88	29.57 ± 29.354	26, 0–268	15.78 ± 36.208	7, 0–339	** < 0.001**
* p*	** < 0.001**		0.094		0.092		**0.005**		
Unspecified									
Yes	19.55 ± 58.440	4, 0–339	7.25 ± 13.175	1, 0–27	16.00 ± 8.192	17.5, 3–26	24.00 ± 77.120	0, 0–339	0.059
No	22.26 ± 25.320	17, 0–268	17.60 ± 18.129	14, 0–88	31.71 ± 31.127	26, 0–268	15.83 ± 20.180	10, 0–105	** < 0.001**
* p*	**0.002**		0.155		**0.016**		0.106		
Treatment									
TTI	21.96 ± 30.653	16, 0–339	17.11 ± 18.000	13, 0–88	30.26 ± 30.082	26, 0–268	17.30 ± 37.043	7, 0–339	** < 0.001**
Surgery	17.94 ± 26.250	13, 0–339	13.31 ± 11.632	11, 0–40	25.24 ± 16.710	24, 0–78	13.81 ± 3 8.732	0.5, 0–339	** < 0.001**
Chemo-radiation	45.67 ± 42.403	34, 1–268	37.85 ± 30.008	31, 1–88	66.92 ± 65.007	43, 26–268	35.41 ± 18.611	33.,8–76	0.137
Death/last follow-up	1663.91 ± 1388.243	1399.5, 3–5767	2192.75 ± 1738.943	1942, 7–5767	1785.31 ± 1362.809	1399.5, 29–4133	1110.69 ± 775.255	1139, 3–2223	** < 0.001**
HNC recurrence/last follow-up	1413.67 ± 1358.980	959.5, 3–5757	1893.43 ± 1712.027	1523, 7–5757	1400.60 ± 1357.374	760, 29–4133	1032.41 ± 811.557	863, 3–2223	**0.003**

*TTI* Time to treatment initiation, *CCI* Charlson comorbidity index, *SD* Standard deviation

Significant p-values (p<0.05) in bold

### Overall survival

**Fig. 2:**
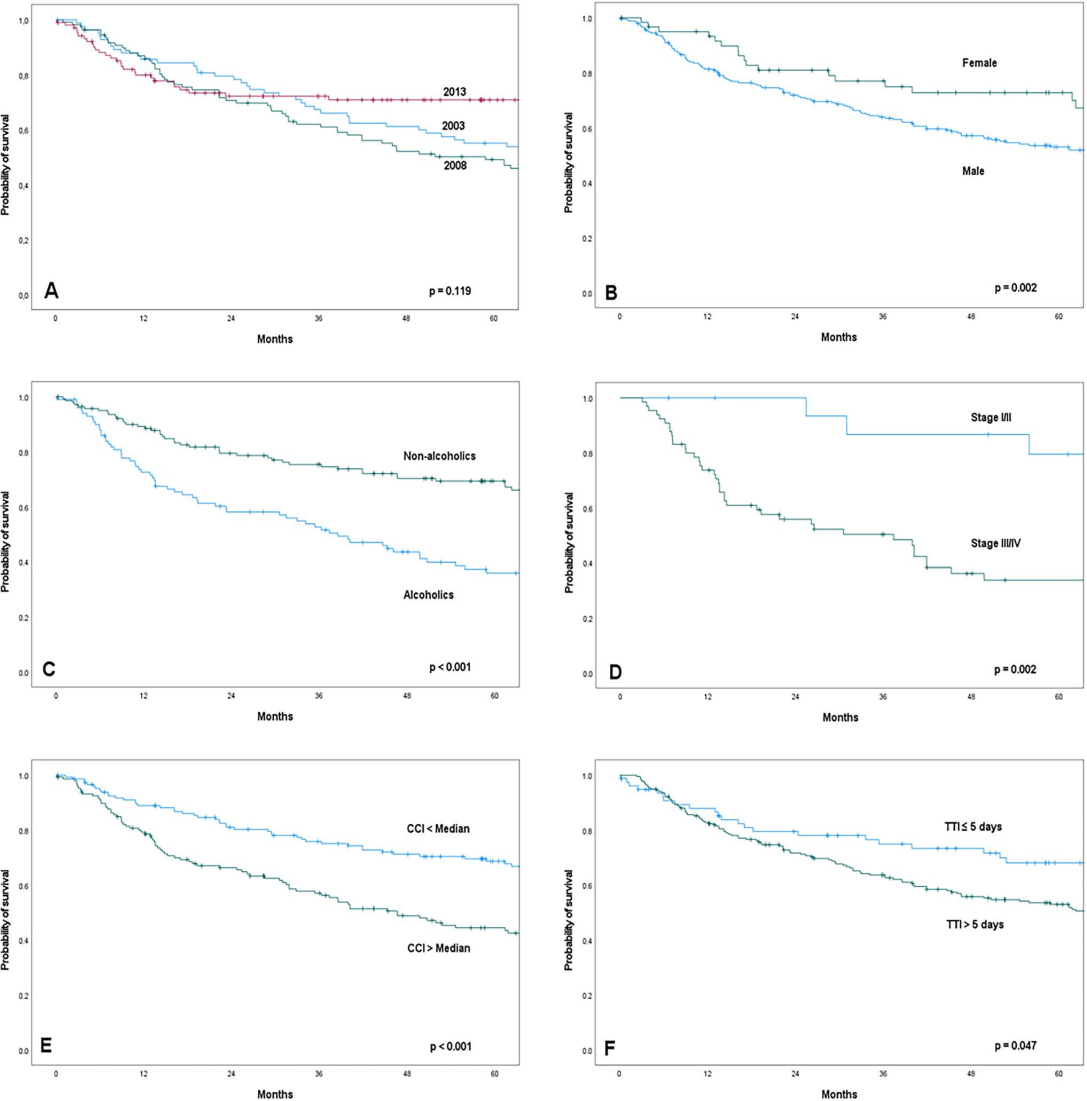
Kaplan–Meier curves of overall survival according to cohort (**A**), gender (**B**), alcoholism (**C**), UICC stage (**D**), Charlson Comorbidity Index (CCI) (**E**) and time to treatment initiation (TTI) (**F**)

The univariable analysis (Supplementary Table S1) showed that men had lower OS than woman (*p = *0.002; Fig. 2). Alcoholic drinking patients (*p <  *0.001) and smokers (*p = *0.002) had also lower OS. A CCI greater than the median also showed a lower survival probability (*p <  *0.001). OS was significantly higher if the age of HNC diagnosis was below the median than above the median (*p = *0.029). HNC patients treated in 2013 showed lower OS (*p = *0.013). The estimated two-year OS was 73.7% and the five-year OS was 56.7%. Overall, the different cohorts showed no significant effect on OS (*p = *0.119). Additionally, patients with TTI > 5 days showed lower OS than patients with TTI ≤ 5 days (*p = *0.047). Significantly longer survival was seen after performing surgical treatment in all cohorts (*p <  *0.001). Patients with a clinical T1/2 tumor showed significantly higher OS than patients with a clinical T3/4 Tumor (*p <  *0.001). Patients who had distant metastases (M1) showed lower OS than patients without distant metastases (M0; *p <  *0.001). Clinical UICC stage I/II was associated with better survival than advanced UICC stage III/IV (*p = *0.002). This result was only seen in the cohort of 2008 (*p = *0.005), while the cohorts of 2003 and 2013 did not show higher OS for UICC stage I/II. Among HNC localizations, HNC of the cavity of the mouth (*p = *0.036) and hypopharynx (*p <  *0.001) was associated with a significant lower OS. Overall, significant influence of the different variables (gender, CCI, age, clinical T, clinical UICC and hypopharynx) on OS was more seen in the cohort of 2008 than in the cohorts of 2003 and 2013. Overall, there was no clear significant influence on OS between the different cohorts (Fig. 2).

Multivariable analyses (Table 3) were performed for all variables influencing OS significantly in the univariable analysis. Men had a 2.3-fold increased hazard of death than women (HR 2.342; 95% CI 1.229–4.466; *p = *0.010). HR for alcohol consumption was 2.054 indicating that HNC patients are more than twice likely to die from alcohol drinking than HNC patients without alcoholism (HR 2.054; 95% 1.319–3.197; *p = *0.002). Increased age at diagnosis (HR 1.026; 95% CI 1.008–1.045; *p = *0.005] and higher CCI (HR 1.109; 95% CI 1.049–1.173; *p = *0.001) showed a slightly increased hazard of death. When the different cohorts were considered as additional parameter, similar results emerged. The year itself did not show a significant influence. The presence of distant metastases (M1) showed a 5.8-fold increased hazard of death than patients without distant metastases (M0) (HR 5.823; 95% CI 2.252–15.058; *p = *0.003). Within the cohorts of 2003 and 2013, some factors had significant impact on OS not seen in the cohort of 2008. Patients of 2013 had 0.3-fold increased hazard of death compared to patients of 2003 (HR 0.327; 95% CI 0.139–0.765; *p = *0.010). Oral cavity tumors had a 1.7-fold increased hazard of death (HR 1.712; CI 1.101–2.661; *p = *0.017) and a hypopharynx tumor had a 2.5-fold increased hazard of death (HR 2.508; 95% CI 1.571–4.003; *p <  *0.001). A TTI lower or higher the median had no impact on OS (HR 1.236; 95% CI 0.852–1.791; *p = *0.264), but TTI ≤ 5 days still showed an effect on OS (HR 1.591; 95% CI 0.997–2.537; *p = *0.051).

**Table 3:** Multivariable analysis of patients’ characteristics, histopathology characteristics and treatment characteristics on OS

Parameter	HR*	Lower 95% CI	Upper 95% CI	*p*
Multivariable analysis I—patients’ characteristics				
Gender				**0.010**
Female	1			
Male	2.342	1.229	4.466	
Alcohol drinking				**0.001**
No	1			
Yes	2.054	1.319	3.197	
Cigarette smoking				0.492
No	1			
Yes	1.182	0.734	1.902	
Age	1.026	1.008	1.045	**0.005**
CCI	1.109	1.049	1.173	** < 0.001**
Year				
2003	1			0.124
2008	1.399	0.881	2.223	0.155
2013	0.916	0.538	1.560	0.747
Multivariable analysis II—histopathology characteristics				
T classification				0.112
T1/2	1			
T3/4	2.157	0.836	5.566	
N classification				0.585
N0	1			
N1/2/3	0.800	0.360	1.781	
M classification				** < 0.001**
M0	1			
M1	5.823	2.252	15.058	
Cancer stage				0.187
Stage I/II	1			
Stage III/IV	2.648	0.623	11.249	
Year				
2003	1			**0.025**
2008	0.787	0.382	1.624	0.517
2013	0.327	0.139	0.765	**0.010**
Multivariable analysis III**—**localization characteristics				
Cavity of the mouth				**0.017**
No	1			
Yes	1.712	1.101	2.661	
Hypopharynx				** < 0.001**
No	1			
Yes	2.508	1.571	4.003	
Year				
2003	1			0.302
2008	1.027	0.705	1.497	0.890
2013	0.734	0.465	1.160	0.185
Multivariable analysis IV—treatment characteristics				
Surgery				** < 0.001**
No	1			
Yes	0.341	0.211	0.551	
Chemo-radiation				0.920
No	1			
Yes	1.024	0.644	1.628	
Year				
2003	1			0.150
2008	0.929	0.646	1.337	0.693
2013	0.646	0.413	1.013	0.057
Multivariable analysis V—waiting time characteristics				
Time to treatment initiation				0.264
< Median	1			
> Median	1.236	0.852	1.791	
First visit to a head neck cancer center				0.203
< Median	1			
> Median	0.797	0.561	1.130	
Year				
2003	1			0.148
2008	0.804	0.546	1.185	0.270
2013	0.644	0.410	1.011	0.056
Multivariable analysis VI—waiting time characteristics, alternative version				
Time to treatment initiation				0.051
< 5 days	1			
> 5 days	1.591	0.997	2.537	
First visit to a head neck cancer center				0.179
< Median	1			
> Median	0.795	0.569	1.111	
Year				
2003	1			0.259
2008	0.796	0.546	1.159	0.234
2013	0.705	0.444	1.117	0.137

*CCI* Charlson comorbidity index, *CI* Confidence interval, *HR* Hazard ratio

Significant p-values (p<0.05) in bold

## Discussion

In this retrospective study, median TTI was 16 days. TTI > 5 days showed significantly lower OS in univariable statistics but not in multivariable statistics. A significantly higher TTI was seen in alcoholism, smoking, patients with combined radio-chemotherapy as primary or adjuvant therapy, higher clinical T stage and cancer of the oropharynx, hypopharynx and esophagus. Significantly lower TTI was seen in patients undergoing surgery, tumors of the parotid gland, facial skin, ear and unspecified HNC. Alcohol has been shown to be a risk factor for HNC of oral cavity and pharyngeal tumors [9]. Alcoholism had a significant impact on waiting time, with a median TTI of 21 days for alcoholics and 15 days for non-alcoholics. Alcoholism can negatively influence compliance and missed appointments can lead to a prolongation of TTI. Cigarette smoking patients showed similar results to alcohol drinking patients. Alcohol drinking patients and smoking patients have in general more comorbidity. Therefore, alcohol and smoking could also have an additional effect via the comorbidity of the patients on TTI. This was not analyzed in this study. A high proportion of stage III/IV was seen. These results are in line with the results of the current literature [1, 3, 10–12]. It has been suggested that delays are related to lack of awareness of symptoms, the patient's own perception of risk and other psychosocial barriers to treatment in time. In the literature, median TTI varies from 20 to 48 days [3, 6, 10, 11, 13–18]. The median TTI of 16 days in this study was shorter. TTI was found to be significantly higher in 2008 than in 2003 and 2013, while there was no continuous trend over the period. In the study of Murphy et al. TTI increased from 19 days in 1998 to 30 days in 2011 [6]. Murphy et al. suggested that the increase in TTI is due to the pursuit of better care, advances in treatment and referral to high-volume centers. Academic facilities are disproportionately more affected to care transitions than comprehensive community health centers. An increase in complexity of treatment (improved surgical reconstruction, preoperative computer-guided reconstruction planning, increase in planning of intensity-modulated radiation) leads to a rising TTI [19]. Lyhne et al. showed that the diagnostic interval was reduced from 20 days in 1992 to 17 days in 2002 and to 13 days in 2010 [10]. In addition, a reduction in waiting times for radiotherapy in Denmark was achieved by the expansion and investment in radiotherapy facilities as a result of the Danish cancer control plans. The introduction of a fast-track system is also believed to have shortened TTI [10]. In a population-based study of 21,623 patients with oral cavity squamous cell carcinoma of the Taiwan Cancer Registry Database, a TTI of less than 30 days was associated to a better survival rate than a TTI of more than 30 days [11]. Surgery was the most common form of therapy with 93.1% in Taiwan. Patients who received primary radiotherapy or chemotherapy tended to have a longer TTI than patients who underwent primary surgery treatment [11].

The geographic regions and medical care are of varying quality and availability in-between the studies. In our study, most examinations were performed directly in the tertiary hospital. In other countries or other health care systems, further examinations may need a referral to another specialists outside the treating center. Treatment organization will be more difficult and requires more time. This may have an impact on TTI. Additionally, the different tumor localizations are not fully comparable. Most studies included a surgical treatment, radiotherapy and chemo-radiation. When only surgery was considered as primary therapy, median TTI was 13 days in the present study. Bilimoria et al. reported a significantly higher median TTI of 23 days for surgical treatment [20]. The waiting time for chemo-radiation as primary therapy was 45.7 days, more than double of the time of surgical treatment. The median was 34 days, similar to Bilimoria et al. with 31 days or Dahlke et al. with 34 days [20, 21]. Primary chemoradiation requires more preparation and organization. In the literature, TTI for primary chemoradiation or primary radiotherapy varies from 31 to 57 days [6, 15, 21–24].

Patients with a TTI greater than the median versus patients with a TTI less than the median showed no significant difference in OS. In contrast, patients with TTI > 5 days showed lower OS than patients with TTI ≤ 5 days in univariable statistics (*p = *0.047) but only a trend in multivariable statistics (*p = *0.051). Anyway, a TTI ≤ 5 days was reached in one quarter of the patients (27%). Van Harten et al. showed that the year of diagnosis is related significantly to treatment delay. Median TTI increased from 31 days between 1990 and 1994 to 38 to 41.5 days in the following periods (1995–1999, 2000–2004, 2005–2010) [25]. In another study by van Harten et al., TTI above the median of 37 days showed a significantly higher HR than waiting less than 37 days [3]. TTI of 61–90 days showed a higher mortality risk than a TTI less than 30 days [6]. In Tsai et al., OS was lower with a waiting time of more than 120 days versus a waiting time of less than 30 days [11]. In Xiao et al., HR was higher with a TTI after ≥ 70 days compared to TTI under 70 days [4]. In Polesel et al. 5-year OS decreased from 62% when waiting time was less than 30 days to 39% when waiting time was more than 90 days [17]. In Schutte et al. the 5-year OS was 78% for TTI up to 30 days and 58% for TTI above 30 days [26]. However, Morse et al. showed different results. In a multi-institution retrospective analysis of 33 819 cases of laryngeal squamous cell cancer (LSCC) based on the National Cancer Database (NCDB) from 2004 to 2013, TTI of 28 days in surgical patients was shown to be not associated with poorer OS in the different tumor localizations (cavity of the mouth, oropharynx, salivary glands and hypopharynx) [16]. TTI of 33 days in non-surgical patients and radiation delay were found to have a significant influence on OS. In contrast, Su et al. were able to show in their study from 2004 to 2009 that a TTI of > 6 weeks has a significant influence on OS. In the present study TTI > 5 days showed a significant lower OS, but only in univariate statistics.

Median TTI of 2003, 2008 and 2013 in this study was 13, 26 and 7 days. TTI was relatively short in all three cohorts. This might explain that small differences did not influence OS between the three cohorts.

Retrospective studies have limitations. The socioeconomic situation of the patients was not considered in this study. This is also a limiting factor, as socioeconomic status may also affect OS and TTI [27]. Furthermore, it might be that TTI was shorter in such patients who brought along relevant diagnostics at first presentation in the hospital. Information on brought-along diagnostics was not available. Furthermore, the retrospective design did not allow to analyze the patient’s influence on TTI (for instance, by non-compliance to appointment). Only in the case with a TTI of 339, this could be retraced to an initial therapy denial.

In literature, TTI was investigated to enable a reduction in waiting times. A first approach is the implementation of a fast-track and well-structured multidisciplinary appointment program. OS for HNC patients can be increased by reducing the time needed for patient referral and an early start of treatment [22]. Furthermore, the implementation of fast-track program has been shown to reduce TTI [28]. In Denmark, a fast-track system was introduced in 2007. TTI reduced from 47 days in 2002 to 25 days in 2010. In the Netherlands, a multidisciplinary first-day consultation (MFDC) was introduced in 2007. The MFDC shall establish a preliminary diagnostic plan and determine the diagnostic procedures in a multidisciplinary consultation from the departments of ear, nose and throat (ENT), oral and maxillofacial surgery, radiotherapy and special dental care. Patients are informed of their diagnostic plan at the end of the day. Van Huizen et al. evaluated the impact of MFDC on TTI and its compliance to Dutch health expectations to start treatment within 30 calendar days. TTI could be reduced with 8 days after 1 year of implementation of MFDC. Furthermore, 83% of patients received first treatment within 30 days instead of 52% before implementation of MFDC [29]. Schutte et al. described a fast-track program and showed a reduction of the median of specialist-to-diagnosis interval from 9 to 2 days and a reduction of TTI from 25 to 18 days [26]. 3-year OS was significantly higher for patients in the new system (84% vs. 72%). Such systems have been shown to increase efficiency in the diagnostic algorithm. To prevent delays in treatment, such a system could also be introduced in Germany. Especially HNC patients with significantly higher TTI in our study could benefit from a fast-track and multidisciplinary appointment program.

According to our analysis, longer TTI > 5 showed negative influence on OS of HNC patients. However, the present data analyses do not contradict the current literature. The findings of the present study need to be verified by further analyses in a prospective study.

## Conclusion

This study investigated the impact of TTI on OS in a tertiary university hospital comparing three HNC patient cohorts from 2003, 2008, and 2013. TTI > 5 days showed lower OS in univariable but not in multivariable analysis. Overall, there was no clear trend in the impact of TTI on OS from the different cohorts. Mean numbers of TTI of 2003, 2008 and 2013 were 17.11, 30.26 and 17.30 days and showed no influence on OS of HNC. Overall, the waiting time in this study was very short with a median of 16 days. TTI was significantly influenced by variables, such as alcoholism, smoking, T classification and tumor localization. However, the findings of the present study need to be verified by further analyses in a prospective study.

## Supplementary Information

Below is the link to the electronic supplementary material.

Supplementary file1 (DOCX 102 KB)
