# Supplementary material for: No impact of time to treatment initiation for head and neck cancer in a tertiary university center in 2003, 2008 and 2013
Source: Eur Arch Otorhinolaryngol. 2022 Apr 30;279(9):4549–60. doi: 10.1007/s00405-022-07392-w (PMC9363340; doi:10.1007/s00405-022-07392-w)
Supplement: Supplementary file 1 — Supplementary file1 (DOCX 102 KB) [file 405_2022_7392_MOESM1_ESM.docx]

**SUPPLEMENTARY TABLES**

**Supplementary Table S1**

| **Supplementary Table S1**. Univariable predictors for overall survival (OS) in relation to patients’ characteristics, histopathology characteristics and treatment characteristics | | | | | | | | | | | | |
| --- | --- | --- | --- | --- | --- | --- | --- | --- | --- | --- | --- | --- |
|  | **All years** | | | **2003** | | | **2008** | | | **2013** | | |
| **Parameter** | **2-Year OS** | **5-Year OS** | **p** | **2-Year OS** | **5-Year OS** | **p** | **2-Year OS** | **5-Year OS** | **p** | **2-Year OS** | **5-Year OS** | **p** |
| **Gender** | | | | | | | | | | | | |
| Male | 0.718 | 0.529 | **0.002** | 0.805 | 0.537 | 0.317 | 0.653 | 0.422 | **0.004** | 0.710 | 0.692 | 0.342 |
| Female | 0.809 | 0.728 |  | 0.727 | 0.636 |  | 0.909 | 0.760 |  | 0.755 | 0.755 |  |
| **Alcohol drinking** | | | | | | | | | | | | |
| Yes | 0.581 | 0.359 | **<0.001** | 0.714 | 0.429 | 0.078 | 0.533 | 0.282 | **0.002** | 0.480 | 0.432 | **<0.001** |
| No | 0.794 | 0.693 |  | 0.778 | 0.778 |  | 0.766 | 0.574 |  | 0.828 | 0.828 |  |
| **Cigarette smoking** | | | | | | | | | | | | |
| Ja | 0.691 | 0.489 | **0.002** | 0.761 | 0.552 | **0.036** | 0.664 | 0.393 | 0.203 | 0.658 | 0.629 | 0.137 |
| Nein | 0.768 | 0.696 |  | 0.889 | 0.889 |  | 0.732 | 0.581 |  | 0.781 | 0.781 |  |
| **CCI** | | | | | | | | | | | | |
| <Median | 0.810 | 0.686 | **<0.001** | 0.818 | 0.636 | 0.122 | 0.825 | 0.622 | **<0.001** | 0.790 | 0.790 | 0.102 |
| >Median | 0.663 | 0.444 |  | 0.780 | 0.493 |  | 0.566 | 0.290 |  | 0.641 | 0.605 |  |
| **Age** | | | | | | | | | | | | |
| <Median | 0.764 | 0.605 | **0.029** | 0.774 | 0.585 | 0.055 | 0.796 | 0.570 | **0.022** | 0.714 | 0.714 | 0.921 |
| >Median | 0.709 | 0.528 |  | 0.833 | 0.486 |  | 0.628 | 0.379 |  | 0.728 | 0.703 |  |
| **Year** | | | | | | | | | | | | |
| All years | 0.737 | 0.567 |  |  |  |  |  |  |  |  |  |  |
| 2003 | 0.795 | 0.550 | 0.119 |  |  |  |  |  |  |  |  |  |
| 2008 | 0.707 | 0.491 |  |  |  |  |  |  |  |  |  |  |
| 2013 | 0.722 | 0.708 |  |  |  |  |  |  |  |  |  |  |
| **T classification** | | | | | | | | | | | | |
| T1/T2 | 0.927 | 0.738 | **<0.001** | - | 0.600 | 0.070 | - | 0.889 | **0.001** | 0.762 | 0.762 | 0.259 |
| T3/T4 | 0.511 | 0.309 |  | 0.563 | 0.250 |  | 0.285 | 0.114 |  | 0.646 | 0.574 |  |
| **N classification** | | | | | | | | | | | | |
| N0 | 0.823 | 0.557 | **0.052** | 0.900 | 0.400 | 0.759 | 0.755 | 0.503 | 0.088 | 0.818 | 0.818 | 0.200 |
| N1.2.3 | 0.580 | 0.408 |  | 0.650 | 0.400 |  | 0.450 | 0.250 |  | 0.631 | 0.573 |  |
| **M classification** | | | | | | | | | | | | |
| M0 | 0.840 | 0.474 | **<0.001** | 0.700 | 0.400 | - | 0.611 | 0.396 | **0.004** | 0.737 | 0.694 | **<0.001** |
| M1 | 0.182 | 0.091 |  | - | - |  | 0.250 | 0.000 |  | 0.143 | 0.143 |  |
| **Cancer stage** | | | | | | | | | | | | |
| stage I/II | - | 0.794 | **0.002** | - | 0.500 | 0.548 | - | 0.857 | **0.005** | - | - | 0.089 |
| stage III/IV | 0.558 | 0.337 |  | 0.714 | 0.286 |  | 0.388 | 0.194 |  | 0.612 | 0.551 |  |
| **Localization** | | | | | | | | | | | | |
| **Cavity of the mouth** | | | | | | | | | | | | |
| Yes | 0.629 | 0.389 | **0.036** | 0.800 | 0.500 | 0.991 | 0.590 | 0.354 | 0.138 | 0.500 | 0.500 | **0.035** |
| No | 0.752 | 0.594 |  | 0.795 | 0.571 |  | 0.729 | 0.518 |  | 0.741 | 0.726 |  |
| **Oropharynx** | | | | | | | | | | | | |
| Yes | 0.851 | 0.632 | 0.339 | 0.833 | 0.556 | 0.506 | 0.881 | 0.641 | 0.106 | 0.824 | 0.741 | 0.424 |
| No | 0.706 | 0.550 |  | 0.784 | 0.549 |  | 0.651 | 0.442 |  | 0.700 | 0.700 |  |
| **Nasopharynx** | | | | | | | | | | | | |
| Yes | 0.600 | 0.600 | 0.659 | 0.333 | 0.333 | 0.711 | - | - | **-** | - | - | 0.248 |
| No | 0.739 | 0.566 |  | 0.812 | 0.558 |  | 0.707 | 0.491 |  | 0.716 | 0.701 |  |
| **Hypopharynx** | | | | | | | | | | | | |
| Yes | 0.471 | 0.354 | **<0.001** | 0.714 | 0.500 | 0.230 | 0.000 | 0.000 | **<0.001** | 0.600 | 0.600 | 0.553 |
| No | 0.768 | 0.592 |  | 0.811 | 0.561 |  | 0.772 | 0.537 |  | 0.732 | 0.717 |  |
| **Larynx** | | | | | | | | | | | | |
| Yes | 0.800 | 0.614 | 0.634 | 0.900 | 0.635 | 0.792 | 0.773 | 0.591 | 0.331 | 0.692 | 0.692 | 0.708 |
| No | 0.721 | 0.556 |  | 0.762 | 0.524 |  | 0.689 | 0.464 |  | 0.739 | 0.710 |  |
| **Nose** | | | | | | | | | | | | |
| Yes | - | 0.833 | 0.357 | - | 0.500 | 0.719 | - | - | 0.182 | - | - | 0.443 |
| No | 0.729 | 0.560 |  | 0.790 | 0.552 |  | 0.695 | 0.481 |  | 0.717 | 0.703 |  |
| **Parotid gland** | | | | | | | | | | | | |
| Yes | 0.909 | 0.808 | 0.131 | - | - | 0.087 | - | 0.500 | 0.577 | 0.750 | 0.750 | 0.900 |
| No | 0.730 | 0.562 |  | 0.790 | 0.577 |  | 0.695 | 0.491 |  | 0.720 | 0.706 |  |
| **Submandibular gland** | | | | | | | | | | | | |
| Yes | - | - | 0.224 | - | - | - | - | - | 0.335 | - | - | 0.556 |
| No | 0.735 | 0.564 |  | 0.795 | 0.550 |  | 0.704 | 0.486 |  | 0.719 | 0.705 |  |
| **Ear** | | | | | | | | | | | | |
| Yes | 0.667 | 0.600 | 0.758 | - | 0.667 | 0.707 | 0.000 | 0.000 | 0.084 | 0.700 | 0.700 | 0.811 |
| No | 0.741 | 0.565 |  | 0.787 | 0.546 |  | 0.720 | 0.500 |  | 0.727 | 0.711 |  |
| **Facial skin** | | | | | | | | | | | | |
| Yes | 0.870 | 0.666 | 0.177 | - | 0.750 | 0.340 | 0.750 | 0.333 | 0.550 | 0.909 | 0.909 | 0.120 |
| No | 0.725 | 0.558 |  | 0.785 | 0.540 |  | 0.703 | 0.503 |  | 0.697 | 0.682 |  |
| **Thyroid** | | | | | | | | | | | | |
| Yes | 0.500 | 0.500 | 0.313 | 0.000 | 0.000 | **<0.001** | 0.500 | 0.500 | 0.978 | 0.667 | 0.667 | 0.331 |
| No | 0.742 | 0.569 |  | 0.805 | 0.594 |  | 0.710 | 0.490 |  | 0.724 | 0.710 |  |
| **Paranasal sinus** | | | | | | | | | | | | |
| Yes | 0.500 | 0.500 | 0.892 | 0.000 | 0.000 | **0.042** | - | - | - | - | - | 0.556 |
| No | 0.738 | 0.568 |  | 0.805 | 0.557 |  | 0.707 | 0.491 |  | 0.719 | 0.705 |  |
| **Oesophagus** | | | | | | | | | | | | |
| Yes | 0.267 | 0.267 | 0.233 | 0.000 | 0.000 | 0.055 | 0.000 | 0.000 | 0.188 | 0.667 | 0.667 | 0.843 |
| No | 0.743 | 0.571 |  | 0.805 | 0.557 |  | 0.713 | 0.496 |  | 0.724 | 0.710 |  |
| **Unspecified** | | | | | | | | | | | | |
| Yes | 0.729 | 0.610 | 0.392 | 0.750 | 0.750 | 0.130 | 0.900 | 0.571 | 0.416 | 0.609 | 0.609 | 0.289 |
| No | 0.738 | 0.563 |  | 0.797 | 0.540 |  | 0.686 | 0.483 |  | 0.743 | 0.726 |  |
| **Treatment** | | | | | | | | | | | | |
| **Surgery** | | | | | | | | | | | | |
| Yes | 0.781 | 0.611 | **<0.001** | 0.847 | 0.593 | **0.006** | 0.755 | 0.544 | **<0.001** | 0.753 | 0.738 | 0.090 |
| No | 0.413 | 0.240 |  | 0.445 | 0.273 |  | 0.333 | 0.167 |  | 0.469 | 0.460 |  |
| **Chemotherapy** | | | | | | | | | | | | |
| Yes | 0.669 | 0.490 | 0.490 | 0.750 | 0.500 | 0.723 | 0.729 | 0.292 | 0.422 | 0.630 | 0.630 | 0.504 |
| No | 0.746 | 0.577 |  | 0.810 | 0.553 |  | 0.705 | 0.505 |  | 0.747 | 0.747 |  |
| **Radiation** | | | | | | | | | | | | |
| Yes | 0.720 | 0.548 | 0.408 | 0.838 | 0.595 | 0.578 | 0.644 | 0.439 | 0.358 | 0.675 | 0.641 | 0.284 |
| No | 0.747 | 0.580 |  | 0.760 | 0.514 |  | 0.740 | 0.518 |  | 0.752 | 0.752 |  |
| **Chemoradiation** | | | | | | | | | | | | |
| Yes | 0.612 | 0.381 | **0.040** | 0.455 | 0.273 | **0.013** | 0.556 | 0.259 | **0.018** | - | - | **0.037** |
| No | 0.761 | 0.604 |  | 0.847 | 0.593 |  | 0.759 | 0.587 |  | 0.694 | 0.679 |  |
| **Immunotherapy** | | | | | | | | | | | | |
| Yes | 0.500 | 0.375 | 0.255 | - | - | **-** | 0.000 | 0.000 | 0.067 | 0.517 | 0.429 | 0.203 |
| No | 0.744 | 0.573 |  | 0.795 | 0.550 |  | 0.713 | 0.496 |  | 0.735 | 0.735 |  |
| **Total waiting (days) in relation to characteristic and diagnosis** | | | | | | | | | | | | |
| **Time to treatment initiation** | | | | | | | | | | | | |
| <Median | 0.763 | 0.591 | 0.366 | 0.878 | 0.564 | 0.264 | 0.736 | 0.502 | 0.607 | 0.690 | 0.690 | 0.453 |
| >Median | 0.710 | 0.526 |  | 0.676 | 0.529 |  | 0.697 | 0.459 |  | 0.790 | 0.746 |  |
| **Time to treatment initiation** |  |  |  |  |  |  |  |  |  |  |  |  |
| ≤ 5 days | 0.795 | 0.680 | **0.047** | 0.905 | 0.619 | 0.330 | 0.762 | 0.610 | 0.408 | 0.749 | - | 0.384 |
| > 5 days | 0.717 | 0.529 |  | 0.758 | 0.527 |  | 0.703 | 0.482 |  | 0.700 | 0.673 |  |
| **Surgery** | | | | | | | | | | | | |
| <Median | 0.765 | 0.608 | 0.874 | 0.867 | 0.593 | 0.637 | 0.736 | 0.502 | 0.812 | 0.707 | 0.707 | 0.383 |
| >Median | 0.796 | 0.614 |  | 0.800 | 0.600 |  | 0.778 | 0.571 |  | 0.852 | 0.791 |  |
| **Chemoradiation** | | | | | | | | | | | | |
| <Median | 0.714 | 0.286 | 0.606 | - | 0.250 | 0.379 | - | - | - | 0.333 | 0.333 | 0.522 |
| >Median | 0.429 | 0.307 |  | 0.333 | 0.333 |  | 0.308 | 0.077 |  | 0.684 | 0.684 |  |

TTI – Time to treatment initiation; CCI – Charlson Comorbidity Index; SD – standard deviation
